# SARS-CoV-2 Spike 1 Protein Controls Natural Killer Cell Activation via the HLA-E/NKG2A Pathway

**DOI:** 10.3390/cells9091975

**Published:** 2020-08-26

**Authors:** Daria Bortolotti, Valentina Gentili, Sabrina Rizzo, Antonella Rotola, Roberta Rizzo

**Affiliations:** Department of Chemical and Pharmaceutical Science, University of Ferrara, 44121 Ferrara, Italy; daria.bortolotti@unife.it (D.B.); valentina.gentili@unife.it (V.G.); sabrina.rizzo@unife.it (S.R.); antonella.rotola@unife.it (A.R.)

**Keywords:** SARS-CoV-2, NK cell, NKG2A, HLA-E

## Abstract

Natural killer cells are important in the control of viral infections. However, the role of NK cells during severe acute respiratory syndrome coronavirus 2 (SARS-CoV-2) infection has previously not been identified. Peripheral blood NK cells from SARS-CoV and SARS-CoV-2 naïve subjects were evaluated for their activation, degranulation, and interferon-gamma expression in the presence of SARS-CoV and SARS-CoV-2 spike proteins. K562 and lung epithelial cells were transfected with spike proteins and co-cultured with NK cells. The analysis was performed by flow cytometry and immune fluorescence. SARS-CoV and SARS-CoV-2 spike proteins did not alter NK cell activation in a K562 in vitro model. On the contrary, SARS-CoV-2 spike 1 protein (SP1) intracellular expression by lung epithelial cells resulted in NK cell-reduced degranulation. Further experiments revealed a concomitant induction of HLA-E expression on the surface of lung epithelial cells and the recognition of an SP1-derived HLA-E-binding peptide. Simultaneously, there was increased modulation of the inhibitory receptor NKG2A/CD94 on NK cells when SP1 was expressed in lung epithelial cells. We ruled out the GATA3 transcription factor as being responsible for HLA-E increased levels and HLA-E/NKG2A interaction as implicated in NK cell exhaustion. We show for the first time that NK cells are affected by SP1 expression in lung epithelial cells via HLA-E/NKG2A interaction. The resulting NK cells’ exhaustion might contribute to immunopathogenesis in SARS-CoV-2 infection.

## 1. Introduction

In December 2019, a novel coronavirus was isolated in Wuhan, China [1]. The severe acute respiratory syndrome coronavirus 2 (SARS-CoV-2) was the causative agent for coronavirus disease 2019 (COVID-19). SARS-CoV-2 is a β-coronavirus with a 79.5% sequence homology with SARS-CoV [2,3]. The CoVs have been demonstrated to be able to adapt quickly and cross the species barrier, as happened with SARS-CoV and Middle East respiratory syndrome CoV (MERS-CoV), with resulting epidemics or pandemics. The effect of these infections on humans often leads to severe clinical symptoms and high mortality [3]. The number of SARS-CoV-2-infected cases is more than 22 million, with typical clinical manifestations including fever, cough, diarrhea, and fatigue [4].

Several studies are currently investigating the potential response of the immune system during SARS-CoV-2 infection [5]. It has already been shown that, during the infection, patients develop an uncontrolled immune response and hyperactivation of macrophages and monocytes [6]. This immune dysregulation is associated with an increase in Interleukin-6 (IL-6), neutrophils, natural killer (NK) [7] cells, and reactive protein C (PCR) and in a decrease in the total number of lymphocytes [5,8]. Interestingly, NK cells showed a functional exhaustion with increased NKG2A expression [8]. NK cells are important effectors in antitumor and anti-infection immunity [9]. The activity of NK cells is controlled by activating and inhibitory receptors [10]. The CD94/NK group 2 member A (NKG2A) is a heterodimeric inhibitory receptor expressed by NK cells [11]. It binds to the nonclassical HLA class I molecule (HLA-E), which presents peptides derived from leader peptide sequences of other HLA class I molecules, including HLA-G [12,13,14]. The ligation of the peptide-loaded HLA-E with NKG2A transduces inhibitory signaling through two inhibitory immune-receptor tyrosine-based inhibition motifs, which suppress NK cytokine cytotoxicity and secretion [11,12,15,16]. By now, no data are available on how SARS-CoV-2 might control NK cells. We evaluated the possible role of SARS-CoV-2 spike proteins in modifying NK cell functions.

## 2. Materials and Methods

### 2.1. NK Cells Purification

Human primary NK cells were obtained from the peripheral blood of four healthy blood donors. This study was approved by the “Ferrara Ethics Committee”. All subjects gave written informed consent in accordance with the Declaration of Helsinki.

Primary NK cells were separated from peripheral blood samples using the negative magnetic cell separation (MACS) system (Miltenyi Biotech, Gladbach, Germany) [17]. The analysis of purified cell fraction by flow cytometry with CD3-PerCp-Cy5.5, CD56-FITC moAbs (e-Bioscience, Frankfurt, Germany), demonstrated that the NK cell content was > 90% (Figure S1A).

### 2.2. Viral RNA Detection

RNA extraction was performed by using the MagMAX Viral/Pathigen Nuclei Acid Isolation kit (ThermoFisher, Milano, Italy) according to the manufacturer’s instructions. The RT-PCR was performed with the TaqMan 2019nCoV assay kit v1 (ThermoFisher, Milano, Italy).

### 2.3. Cell Culture

NK cell were supplemented with IL-12 (10 ng/mL) (Becton Dickinson, San Jose, CA, USA) to have a positive control for IFN-gamma secretion [18].

The Beas-2B (ATCC CRL-9609) bronchial epithelial cell line was grown in BEGM culture medium (BEGM Kit Catalog No. CC-3170; Lonza/Clonetics Corporation, USA). The K562 (ATC CCL-243) lymphoblastoid cell line was cultured in Roswell Park Memorial Institute medium (RPMI) (Gibco, Milano, Italy) supplemented with 10% of FCS (fetal calf serum, Euroclone, Pero, MI, Italy) and 100 U/mL penicillin and 100 µg/mL streptomycin. Cell cultures were maintained at 37 °C in a humidified atmosphere of 5% CO_2_ in air.

The role of HLA-E and NKG2A was evaluated by incubating the cells with anti-HLA-E (clone MEM-E/08, Exbio, Praha, CZ) or anti-CD94/NKG2A (clone 131411, BD, Italy) antibodies (7.5 ng/mL).

GATA3 DNA-binding activity was inhibited by adding pyrrothiogatain (cat#sc-352288A, Santa Cruz, CA, USA) to cell cultures (10 μM) [19].

### 2.4. Flow Cytometry

In total, 1 × 10^5^ eNK cells were labeled with fluorophore-conjugated antibodies: CD3-PE (clone SP34-2, CD16-APC (clone B73.1), CD56-PE-Cy7 (clone B159), NKG2A-APC (clone 131411), and matched isotype controls.

In total, 5 × 10^5^ bronchial epithelial cells were stained specific Ab HLA-I (HLA-A, -B, -C)-PE (Becton Dickinson, San Jose, CA, USA), HLA-E (clone MEM-E/08)-FITC, and matched isotype controls.

GATA3 expression was evaluated in fixed and permeabilized cells with IntraPrep reagent (Beckman Coulter; Brea, CA, USA) and stained with anti-GATA3 (BV421; BD)-PE MoAb. CK8 expression was evaluated with anti-CK8 (TS1, Novus Biologicals, Milano, Italy)-PE MoAb.

SP1, SP2, and S proteins expression in K562 and Beas-2B transfected cells were evaluated with anti-spike 1 (clone 2C1), anti-spike 2, and anti Sars-Cov spike antibodies (myBiosource, San Diego, CA, USA).

Data were analyzed using FACS CantoII flow cytometer (BD, Milan, Italy) and FlowJo LLC analysis software (Tree Star Inc, Ashland, OR, USA). In total, 10,000 events were acquired.

### 2.5. Immunofluorescence Assay

HLA-E expression was analyzed by immunofluorescence with anti-HLA-E-PE antibody (MEM-E/08-PE), as previously described [20,21]. In particular, 10^5^ cells were spotted onto glass slides and fixed by cold acetone for 30 min at 20 °C. Slides were air-dried and kept at 20 °C until use. For assay, slides were rehydrated by washings in PBS, and incubated with anti HLA-E-PE-labelled antibody diluted 1:100 in PBS for 35 min at 37 °C in a humidified chamber. Slides were washed twice with PBS for 10 min, once for 1 min with tap water, and once for 1 min with distilled water. After washings as described, slides were stained with 1:2000 in PBS diluted Hoechst stock solution (ThermoFisher, Milano, Italy) for 5–10 min, washed in distilled water, and finally mounted with 50% glycerol in PBS for fluorescence microscope observation. All samples were observed under a UV light microscope (Nikon Eclipse E600) equipped with a digital camera (DMX 1200).

### 2.6. Cell Migration Assay

The assay was performed using the Cornig Transwell System, with inserts with 5-μm pore polycarbonate membrane. Briefly, 1 × 10^6^ cells were seeded in the upper chamber in 150 μL of RPMI with the 0.5% of FBS and IL-2 (100 IU/mL; Sigma Biosciences, MO, USA). The inserts containing cells were positioned into a 24-well plate, which provides the lower reservoirs for the migration system. Each reservoir was filled with 650 μL of medium (RPMI) containing SARS-CoV-2 proteins (spike protein S1 subunit, spike protein S2 subunit) (RayBiotech, Peachtree Corners, GA, USA) or control SARS-CoV spike protein (MyBiosource, San Diego, CA, USA) at the final concentration of 100 ng/mL. Medium with CXCL12 (100 ng/mL; BioLegend, San Diego, CA, USA) [22] was used as the positive. Migration was performed for 3 h at 37 °C and then the plate was briefly centrifuged at 300× *g* for 5 min in order to collect migrated cells in the lower reservoir for the cell count. Every condition was tested in triplicate and results were reported as the number of migrated cells compared to untreated NK cells.

### 2.7. Protein Transfection

K562 or Beas-2B cell lines were transfected using the Pierce Protein Transfection Kit (ThermoFisher, Milano, Italy) following the product instructions. A total of 4 × 10^5^ cells were transfected with 1 μg of protein (spike protein S1 subunit, spike protein S2 subunit) of SARS-CoV-2 or SARS-CoV spike protein. Transfection was performed for 3–4 h at 37 °C in 1 mL of medium without FBS. After transfection, a volume of complete medium with 20% FBS was added to each well. K562 or Beas-2B cells treated with transfection reagent alone or transfected with 0.5 μg of control fluorescent antibody (provided in the kit) were used as the negative and efficiency control, respectively.

### 2.8. Lactate Dehydrogenase (LDH) Assay

The LDH assay was performed to evaluate the effect of the transfection with SARS-CoV-2 and SARS-CoV proteins in K562 or Beas-2B cells on cell viability. Transfected K562 or Beas-2B cells were suspended at 5 × 10^4^ cells/mL and cultured for 4 h on a 96-well microplate at 37 °C with 5% CO_2_. A colorimetric-based lactate dehydrogenase (LDH) assay (Cytotoxicity Detection KitPLUS; Switzerland) was used, according to the manufacturer’s instructions.

### 2.9. Degranulation Analysis

In vitro cytotoxicity experiments were performed using K562 or Beas-2B cells as the target and NK cells as effector cells. NK cells were added to K562 or Beas-2B cells with a 5:1 effector:target ratio [23]. NK cell degranulation was evaluated by CD107a staining (anti-CD107a-PE; clone H4A3; BD Biosciences) after 3 h of treatment with Golgi Stop solution (BD). Labeled cells were analyzed with a FACSCantoII flow cytometer (BD, Milano, Italy) and FlowJo software (Tree Star Inc., Ashland, OR, USA).

### 2.10. Carboxyfluorescein Diacetate Succinimidyl Ester (CFSE) Analysis

K562 or Beas-2B cells were labeled with carboxyfluorescein diacetate succinimidyl ester (CFSE) to assess cell-mediated cytotoxicity, using a 7AAD/CFSE Cell-mediated cytotoxicity assay kit (Ann Arbor, MI, USA). In total, 10^7^ cells/mL were resuspended in CFSE staining solution and incubated for 15 min at 37 °C. Control target cells were resuspended in 0.1% BSA. Then, cells were washed two times with culture medium and incubated for 30 min at 37 °C. NK cells were put in co-culture with CFSE-labeled infected cells at a 1:5 ratio. The cell mixture was incubated for 4 h, centrifuged, and resuspended in 7-AAD staining solution. Control target cells were resuspended in assay buffer. Cells were incubated for 15 min in the dark at 4 °C. Then cells were centrifuged and resuspended in assay buffer. Cells were analyzed with a FACSCantoII flow cytometer and FlowJo software.

### 2.11. IFN-gamma ELISA Assay

IFN-gamma levels were detected by an IFN-gamma ELISA kit (MyBiosource, San Diego, CA, USA) following the instructions. In particular, standards and samples were pipetted into the wells and IFN gamma present in a sample was bound to the wells by the immobilized antibody. The wells were washed and biotinylated anti-Human IFN gamma antibody was added. After washing away unbound biotinylated antibody, HRP-conjugated streptavidin was pipetted to the wells. The wells were again washed, a TMB substrate solution was added to the wells, and color developed in proportion to the amount of IFN gamma bound. The Stop Solution changed the color from blue to yellow, and the intensity of the color was measured at 450 nm (ThermoFisher ELISA Reader).

### 2.12. Real-Time PCR

RNA extraction was performed by using the RNeasy kit (Qiagen, Hilden, Germany) according to the manufacturer’s instructions. The Real-Time PCR was performed with the TaqMan 2019nCoV assay TaqMan gene expression assays for HLA- E (Hs00428366_m1), GATA3 (Hs00231122_m1), Eotaxin3 (Hs00171146_m1), and NKG2A (Hs00970273_g1) (ThermoFisher).

### 2.13. HLA-E Binding Prediction

The HLA-E binding prediction was made using the IEDB analysis resource ANN aka NetMHC (ver. 4.0) tool at the http://tools.iedb.org/mhci/help/, using the viral spike 1 protein from the SARS-CoV-2 sequence (QHO62112.1).

### 2.14. Statistical Analysis

Since the biological variables presented a normal distribution (Kruskal–Wallis test, *p* > 0.05), they were evaluated by Student *t* test by Graph Pad Prism 6 software (https://www.graphpad.com/scientific-software/prism/). A *p*-value < 0.05 was defined statistically significant.

### 2.15. Data Availability

All relevant data are within the manuscript.

## 3. Results

### 3.1. Spike Proteins Induce NK Cell Migration

**Figure 1:**
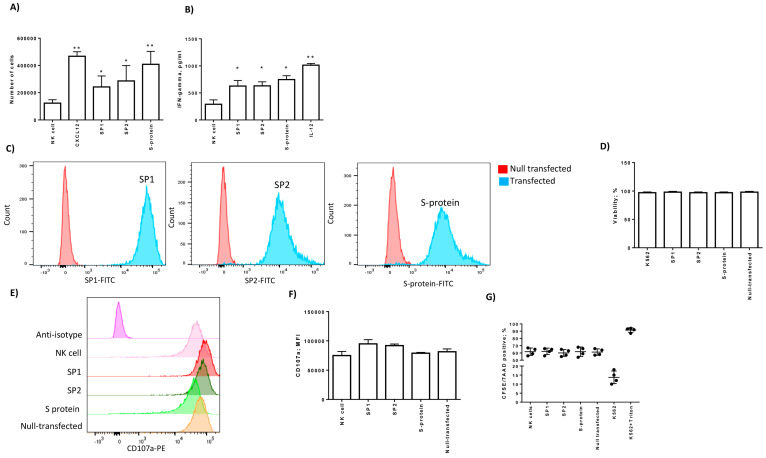
Peripheral blood NK cells from four control subjects negative for both SARS-CoV-2 and SARS-CoV viremia were cultured NK cells in the presence of SP1, SP2 from SARS-CoV-2, or spike protein (S) from SARS-CoV. (**A**) NK cell migration is reported as the cell number. We used CXCL12 as a positive control for NK cell migration. NK cells with no treatment were used as negative control (NK cells). (**B**) Secretion of IFN-gamma evaluated by ELISA. IL-12 treatment was used as the control of IFN-gamma secretion. (**C**) Representative intracellular expression of SP1, SP2, and S proteins in K562 cells after transfection. (**D**) K562 viability, assessed by the LDH assay, in basal and transfected conditions. The transfection with 0.5 µg of control fluorescent antibody was used as a positive control (null-transfected). (**E**) Representative histograms of NK cell degranulation towards the K562 cell target. NK cells were marked with CD56-PECy7 (see Figure S1C). The degranulation was assessed by CD107a-PE expression. (**F**) Mean fluorescence intensity (MFI) of CD107a-positive NK cells after the co-culture with K562 target cells. (**G**) CFSE+/7-AAD+ cell percentage in NK cell/K562 co-cultures. K562 alone and treated with Triton x-100 (0.8%) were used as negative and positive controls, respectively. The values are presented as mean ± standard deviation. *, ** significant *p* values Student *t* test.

Although the role of NK cells in the immune response towards viral infections is generally accepted, there are few data on the early NK cell trafficking in response to coronavirus infections [5]. The evaluation of NK response to SARS-CoV-2 infection is important to determining the innate immune response per se and for the cross-talk with adaptive immune cells [24]. We explored the migration, interferon-gamma (IFN-gamma) secretion, and degranulation of NK cells in the presence of spike 1 (SP1) and spike 2 (SP2) SARS-CoV-2 proteins and SARS-CoV spike proteins (S). We used a cell migration system in a 5-µm transwells w/polycarbonate filter, without a cell barrier. We purified NK cells from the peripheral blood of control subjects (Figure S1A) negative for both SARS-CoV-2 and SARS-CoV viremia (data not shown). This condition ensured that NK cells were naïve for spike proteins. We cultured NK cells in the presence of SP1, SP2 from SARS-CoV-2, or spike protein (S) from SARS-CoV and we observed no effect on cell viability (Figure S1B). We used CXCL12 as a positive control for NK cell migration [22]. We observed an increase in NK cell migration in the presence of both SP1 and SP2 (SP1 *p* = 0.021; SP2 *p* = 0.013 Student *t* test) (Figure 1A). Similarly, NK cell migration was induced by the SARS-CoV S protein (*p* < 0.01; Student *t* test) (Figure 1A). No modifications in cell viability were observed (Figure S1B). These data suggest that both SARS-CoV-2 and SARS-CoV spike proteins are able to chemo-attract NK cells. To evaluate if spike proteins are able to also induce NK cells’ activation, we analyzed the secretion of IFN-gamma. We observed an increase in IFN-gamma secretion in the presence of SP1, SP2, and S-protein (*p* < 0.001; Student *t* test) (Figure 1B). The basal IFN-gamma secretion might be enhanced by the IL-2 treatment of NK cells, which is necessary for the in vitro maintenance of primary NK cells.

### 3.2. Spike Proteins Did Not Modify NK Cell Cytotoxicity

Then, we evaluated the cytotoxicity of NK cells in the presence of spike proteins, using CD107a staining, a marker of degranulation [23]. We mimicked the expression of spike proteins inside NK target cells, the K562 cell line, transfecting directly the proteins. We obtained a mean transfection efficiency of 95% for SP1, SP2, and S proteins (Figure 1C). The K562 cell viability, evaluated by the LDH assay, was not affected by protein transfection (Figure 1D). We incubated NK cells for four hours with K562 and evaluated the expression of CD107a on CD56-gated NK cells (Figure S1C). We observed an increase in CD107a staining in all the culture conditions, with no difference in the presence of SP1, SP2, and S proteins in comparison with the untreated co-culture (S1 *p* = 0.078; S2 *p* = 0.087; S *p* = 0.081; Student *t* test) (Figure 1E,F). To be sure that NK cells expressing CD107a were able to kill K562 cells, CFSE (carboxyfluorescein diacetate succinimidyl ester), the staining of target cells was detected by flow cytometry [20]. We observed an increase in the K562 CFSE+/7-AAD+ cell percentage in all the co-culture conditions comparable with the killing observed in the untreated co-culture (*p* < 0.001; Student *t* test) (Figure 1G), confirming the results observed with CD107a staining (Figure 1E,F).

### 3.3. SARS-CoV-2 Spike 1 Protein Modifies NK Cell Cytotoxicity When Presented by Lung Epithelial Cells

**Figure 2:**
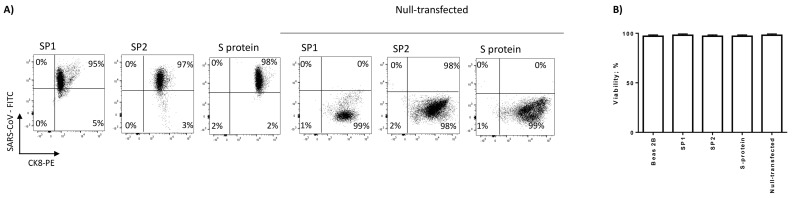
(**A**) Representative intracellular expression of SP1, SP2, and S proteins and surface expression of the epithelial marker CK8-PE in Beas-2B cells after transfection. (**B**) Beas-2B viability, assessed by the LDH assay, in basal and transfected conditions. The transfection with 0.5 µg of control fluorescent antibody was used as a positive control (null-transfected). The values are presented as mean ± standard deviation.

Since the target cells for SARS-CoV-2 replication are lung epithelial cells [4], we considered the activation status of NK cells in this context. We mimicked the expression of SP1, SP2, and S proteins inside Beas-2B lung epithelial cells, transfecting directly the proteins. We obtained an average 95% efficiency of transfection with all the proteins (Figure 2A). The transfection did not affect cell viability (Figure 2B) and the expression of CK8 epithelial cell markers (Figure 2A).

**Figure 3:**
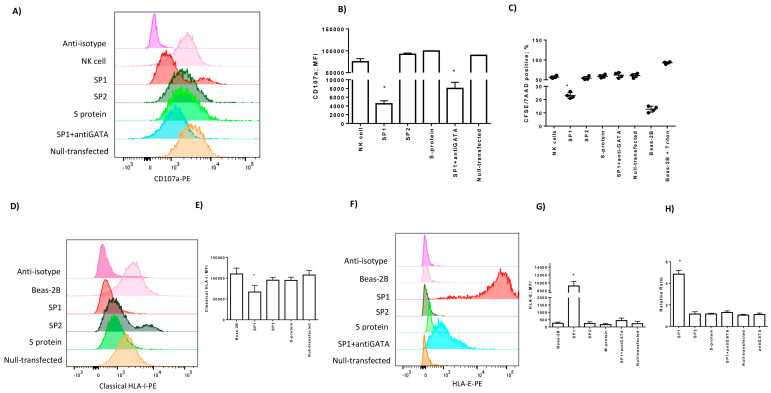
(**A**) Representative histograms of NK cell degranulation towards the Beas-2B cell target. NK cells were marked with CD56 and gated as reported in Figure S1C. The degranulation was assessed by CD107a-PE expression. Beas-2B was transfected with SP1, SP2, and S proteins or control fluorescent antibody was used as the positive control (null-transfected), or treated with GATA3 inhibitor (anti-GATA). (**B**) MFI of CD107a-PE-positive NK cells after the co-culture with Beas-2B cells. (**C**) CFSE+/7-AAD+ cell percentage in NK cell/Beas-2B co-cultures. Beas-2B alone and treated with Triton-x100 (0.8%) were used as negative and positive controls, respectively. The values are presented as mean ± standard deviation. (**D**) Representative histograms of HLA-I expression on the surface of transfected Beas-2B. (**E**) The histograms showed the mean ± standard deviation MFI (mean fluorescence intensity) values of HLA-I expression in three independent experiments. * significant *p* values Student *t* test; (**F**) Representative histograms of HLA-E expression on the surface of transfected Beas-2B cells. (**G**) The histograms showed the mean ± standard deviation MFI (mean fluorescence intensity) values of HLA-E expression in three independent experiments. * significant *p* values *t* test. (**H**) Relative ratio of Real-Time PCR of HLA-E expression in transfected Beas-2B cells. SP1 transfected Beas-2B cells were also treated with GATA inhibitor (anti-GATA). * significant *p* values Student *t* test.

We incubated NK cells for four hours with lung epithelial cells and evaluated the expression of the CD107a degranulation marker. We observed a decrease in CD107a staining in the culture condition with SP1-transfected Beas-2B cells (*p* < 0.001; Student *t* test) (Figure 3A,B). On the contrary, we observed an increase in CD107a expression in the co-culture conditions with SP2 and S protein-transfected Beas-2B cells, which is similar to that observed with the control of the transfection condition (null-transfected) and untreated co-culture (*p* = 0.079; *p* = 0.085, respectively; Student *t* test) (Figure 3A,B).

To be sure that NK cells expressing CD107a were able to kill Beas2B cells, CFSE staining of target cells was detected by flow cytometry. We observed an increase in the Beas-2B CFSE+/7-AAD+ cell percentage in the co-culture with NK cells in SP2 and S protein-transfected Beas2B cells (Figure 3C), while there was a decrease in the Beas-2B CFSE+/7-AAD+ cell percentage in the co-culture with NK cells in SP1-transfected Beas-2B cells (*p* < 0.001; Student *t* test) (Figure 3C), confirming the results observed with CD107a staining (Figure 3A,B).

### 3.4. Spike 1 Peptide Is Presented by Lung Epithelial Cells via HLA-E Molecules

Since the presence of intracellular SP1 protein in lung epithelial cells induced a decrease in the cytotoxic activity of NK cells, we analyzed the possible factors involved in the modification of NK cell status.

**Figure 4:**
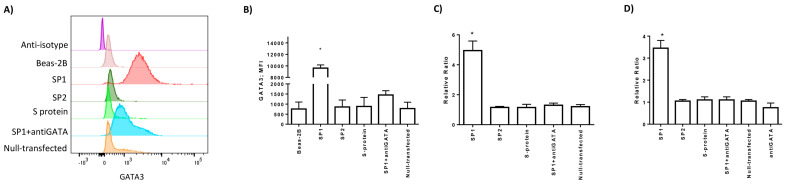
(**A**) Representative histograms of GATA3 expression on the surface of Beas-2B cells after transfection with SP1, SP2, and S proteins or control fluorescent antibody was used as a positive control (null-transfected) or treated with GATA inhibitor (anti-GATA). (**B**) The histograms showed the mean ± standard deviation MFI (mean fluorescence intensity) values of GATA3 expression in three independent experiments. * significant *p* values Student *t* test. (**C**) Relative ratio of real-time PCR of GATA3 expression in transfected Beas-2B cells. SP1-transfected Beas-2B cells were also treated with GATA inhibitor (anti-GATA). (**D**) Relative ratio of real-time PCR of GATA3 target gene Eotaxin3/CCL26 in transfected Beas-2B cells. SP1-transfected Beas-2B cells were also treated with GATA inhibitor (anti-GATA). * significant *p* values Student *t* test.

Since viral proteins are commonly recognized and degraded by the proteasome inside the infected cells [25], we hypothesized that SP1 peptides might be presented to NK cells. Intracellular peptide presentation is performed by human leukocyte antigen (HLA) class I molecules, which are expressed by all nucleated cells. Firstly, we evaluated the expression of HLA class I molecules in Beas-2B cells transfected with SP1, SP2, and S proteins. When we stained the cells with anticlassical HLA class I molecules (HLA-A, HLA-B, HLA-C) antibody, we recognized a significant decrease in their membrane expression when lung epithelial cells were transfected with SP1 protein (*p* < 0.001; Student *t* test) (Figure 3D,E). SP2 and S protein slightly inhibited HLA-I expression (*p* = 0.039; *p* = 0.041, respectively; Student *t* test). Epithelial lung cells can express also non-classical HLA-E molecule [7]. HLA-E binds peptides primarily derived from specific signal sequences [26] and interacts with NKG2A/CD94 NK cell inhibitory receptors [15]. When we performed the lung epithelial cell staining with MEM-E/06 anti-HLA-E antibody, we observed no expression in basal conditions, which was not affected by SP2 and S transfection (Figure 3F,H). On the contrary, there was a significant increase in HLA-E protein (*p* = 0.011; Student *t* test) (Figure 3F,G) and mRNA expression (*p* < 0.001; Student *t* test) (Figure 3H), in the presence of SP1 protein. HLA-E expression is controlled by the GATA3 transcription factor [12], which is known to also be expressed by lung epithelial cells [27,28]. We observed an increase in GATA3 protein (*p* < 0.001; Student *t* test) (Figure 4A,B) and mRNA (*p* < 0.01; Student *t* test) (Figure 4C) expression only in the presence of SP1. To confirm the transcriptional activity of GATA3 induced by SP1, we analyzed the expression levels of a target gene for GATA3, the Eotaxin3/CCL26 [29]. We observed the induction of CCL26 transcription only in the presence of SP1 (*p* < 0.001; Student *t* test) (Figure 4D). The treatment with a GATA inhibitor, the pyrrothiogatain [19], reduced both GATA3 protein (Figure 4A,B) and mRNA (Figure 4C) expression. To be sure that the increase in HLA-E expression in lung epithelial cells transfected with SP1 protein is controlled by GATA3 transcription factor, we treated the cells with pyrrothiogatain. This inhibitory molecule reduced the expression and transcription of the HLA-E gene in Beas-2B cells (Figure 3F,H).

**Figure 5:**
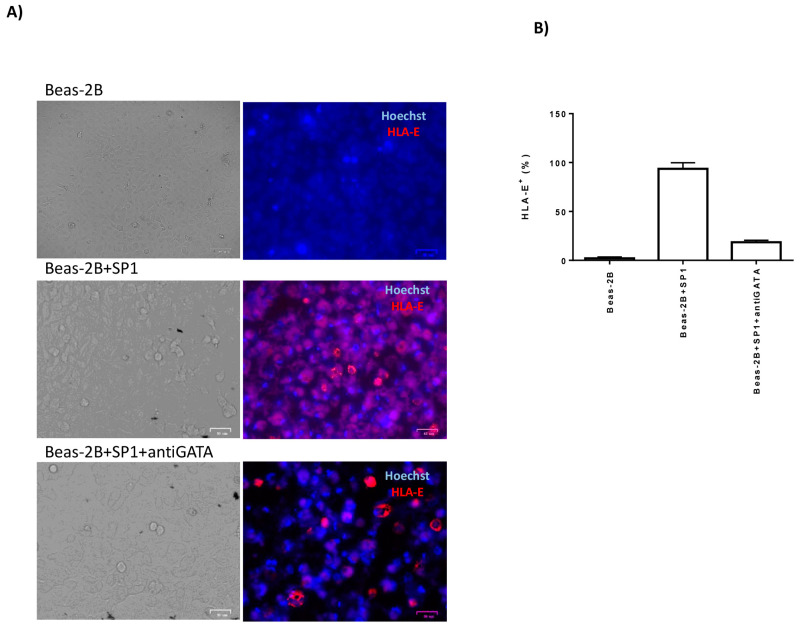
HLA-E expression in Beas-2B cells was characterized by immunofluorescence (Nikon Eclipse TE2000S, equipped with a digital camera). The evaluation was assessed after SP1 transfection (SP1) without or with GATA inhibitor (antiGATA inhibitor). (**A**) The cells were stained with Hoechst for nuclear detection and anti-HLA-E-PE monoclonal antibody (clone MEM-E/08, Exbio, Praha, Czech Republic). Original magnification 40×. (**B**) Percentage of HLA-E positive (+) cells in the Beas-2B and after SP1 transfection (SP1) without or with GATA inhibitor (antiGATA).

The immune-fluorescence staining showed increased modulation of the expression of HLA-E molecules in the presence of intracellular SP1, which was reduced with the addition of GATA inhibitor (Figure 5A,B). To evaluate if SP1 peptides might be presented by HLA-E molecules, we performed MHCI (Major histocompatibility Class I) binding predictions, made on 26 April 2020 using the IEDB analysis resource ANN aka NetMHC (ver. 4.0) tool. We observed that an 8mer peptide in the SP1 domain (270-277, LQPRTFLL) showed a high affinity mainly for the HLA-E*0101 binding groove (IC50: 0.02) and in a lower extent for the HLA-E*0301 allele (IC50: 0.76), with a similar consensus motif with HLA-E binding (VMAPRTVLL) [14]. We might speculate that the induction of GATA3 and the binding of SP1 peptide might induce HLA-E membrane expression on lung epithelial cells.

### 3.5. HLA-E/NKG2A/CD94 Interaction Is Responsible for NK Cells’ Exhaustion

**Figure 6:**
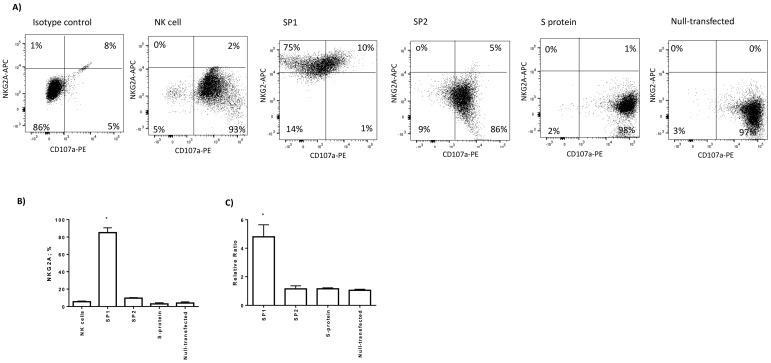
(**A**) Representative dot plots of NKG2A/CD94 and CD107a expression on the surface of NK cells after co-culture with Beas-2B transfected with SP1, SP2, and S proteins or control fluorescent antibody was used as a positive control (null-transfected). (**B**) The histograms show the mean ± standard deviation percentage of NKG2A/CD94-positive cells in three independent experiments. * significant *p* values Student *t* test. (**C**) Relative ratio of real-time PCR of NKG2A expression in transfected Beas-2B cells. * significant *p* values Student *t* test.

Since the functional control of NK cells by HLA-E is possible in the presence of NKG2A/CD94 on NK cells, we evaluated the expression of this receptor on the surface of NK cells. When NK cells were co-cultured with SP1-transfected Beas-2B cells, we observed an increase in the protein (*p* < 0.001; Student *t* test) (Figure 6A,B) and mRNA (*p* < 0.01; Student *t* test) (Figure 6C) expression of the inhibitory receptor NKG2A/CD94. Interestingly, the percentage of NKG2A-positive NK cells increased from an average of 16% to 80%. Interestingly, a high percentage of NKG2A-positive NK cells were also CD107A negative (Figure 6A).

**Figure 7:**
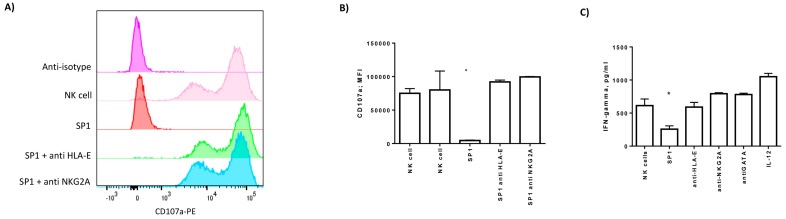
(**A**) Representative histograms of NK cell degranulation towards Beas-2B cells transfected with SP1 protein without or with anti-HLA-E, anti-NKG2A, or anti-isotype control. NK cells were marked with CD56 and gated as reported in Figure S1C. The degranulation was assessed by CD107a-PE expression. (**B**) MFI of CD107a in NK cells after the co-culture with Beas-2B cells transfected with SP1 protein without or with anti-HLA-E, anti-NKG2A, or anti-isotype control. The values are presented as mean ± standard deviation. * significant *p* values Student *t* test. (**C**) Secretion of IFN-gamma evaluated by ELISA. IL-12 treatment was used as a control of IFN-gamma secretion.

To be sure that the resulting inactivity of NK cells towards lung epithelial cells expressing SP1 was determined by HLA-E/NKG2A interaction, we treated the cell culture with anti-HLA-E or anti-NKG2A/CD94 antibodies. We incubated NK cells for four hours with the SP1-transfected Beas-2B cell line and evaluated the expression of CD107a in the presence or absence of anti-HLA-E or anti-NKG2A antibodies. We observed a decrease in CD107a-positive NK cells in the culture condition with SP1-transfected Beas-2B cells (*p* < 0.001; Student *t* test) (Figure 7A,B), which was recovered by the treatment with anti-HLA-E and anti-NKG2A/CD94 antibodies (Figure 7A,B). As a proof of concept, the secretion of IFN-gamma was reduced by SP1 treatment (*p* < 0.01; Student *t* test) (Figure 7C), while it was enhanced after the treatment with anti-HLA-E, anti-NKG2A/CD94 antibodies, and GATA inhibitor (Figure 7C).

## 4. Discussion

The gaps in the activation of the immune system during SARS-CoV-2 infection translate into the severity of the COVID19 disease. Recent studies have documented a modification in the NK cell number and phenotype [5,8,30]. The total number of NK cells decreased in patients with SARS-CoV-2 infection and the expression of NKG2A on the surface of NK cells was increased, suggesting an exhausted phenotype [31,32]. Interestingly, when the patients were rescued after the infection, NKG2A expression was decreased simultaneously with the increase in the number of NK cells [30]. These results suggest that SARS-CoV-2 infection might compromise the innate antiviral immunity exhausting NK cells’ functions [33,34].

We evaluated the effect of SARS-CoV-2 spike proteins in the control of NK cell activation. We considered spike 1 protein, which is involved in the attachment of the virion to the cell membrane by interacting with ACE2 receptor [35], and spike 2 protein that mediates the fusion of the virion. We observed that the extracellular spike proteins from SARS-CoV-2 and SARS-CoV are able to induce NK cell chemotaxis and activation, via the induction of IFN-gamma secretion in the K562 cell model. These results are interesting considering the efficacy of IFN-gamma in inhibiting SARS-CoV replication partly through the downregulation of ACE2 [36]. Our data sustain the role of the immune response of NK cells during SARS-CoV-2 infection [37]. They would migrate to the infected sites and respond to viruses producing IFN-gamma, killing virus-infected cells, and boosting the adaptive immune response with the production of innate proinflammatory cytokine and type I IFNs [5]. The spike proteins, per se, are not able to affect NK cell activation and IFN-gamma secretion.

Since SARS-CoV-2 infection is mainly localized to lung epithelial cells, where the detrimental effects of this infection are more evident [4], we evaluated the effect of SARS-CoV proteins on this cell type.

Surprisingly, we showed that the intracellular expression of S1 protein in lung epithelial cells reduces the activation of NK cells and their ability to degranulate, which is the opposite pattern to that observed in K562 cells. These results account for the in vivo observation of a break in the interplay of lung epithelial cells and immune cells during SARS-CoV-2 infection [37], with a consequent exhausted immune response [37].

To evaluate the possible mechanisms used by SARS-CoV proteins to control NK cells’ activation, we considered that the activation of NK cells is partly controlled by the expression of HLA class molecules, via the interaction with specific NK cell receptors [38]. We evaluated the possible modification of surface HLA class I molecules on lung epithelial cells. The presentation of pathogen-derived peptides by HLA molecules and the genetic variability of HLA alleles in human populations account for their role in the individual responses to SARS-CoV-2 infection and/or progression. We showed that S1 protein on one side diminished classical HLA class I molecule expression but on the other side upregulated HLA-E expression. The protein HLA-E is a non-classical major histocompatibility complex molecule that binds peptides derived from a specific signal sequence [14]. We recognized an SP1-derived HLA-E binding peptide that might stabilize HLA-E expression on the surface of lung epithelial cells during SARS-CoV-2 infection. Interestingly, the highest affinity is demonstrated for the HLA-E*0101 allele. HLA-E surface expression conferred cell resistance to NK cell lysis, interacting with the NK cell inhibitory receptor CD94/NKG2A [14,38]. The involvement of HLA-E in the control of NK cell activation is confirmed also by the different results observed in K562 and lung cell models. K562 cells express no HLA molecules, providing no effective molecules to SARS-CoV spike 1 protein in order to control NK cells. For this, we observed IFN-gamma secretion and NK cell activation also in the presence of SARS-CoV spike 1 protein in the K562 cell model. On the contrary, lung cells, which express HLA-E molecules in the presence of SARS-CoV spike 1 protein, are able to inhibit IFN-gamma secretion and NK cell activation.

Since, it was shown earlier that HLA-E is tightly upregulated through GATA3 response elements [12], we evaluated the role of this transcription factor. We observed that *HLA-E* up-modulation by SP1 is controlled by GATA3 transcription factor. In fact, the treatment with GATA inhibitor reduced the expression of HLA-E even in the presence of SP1. GATA3 is a transcription factor that drives the differentiation of T helper (Th) 2 cells [28], immune regulation [27], and embryonic and adult non-hematopoietic cells’ differentiation, including the lung [39]. The upregulation of GATA3 mRNA and protein by SP1 protein might have other important effects on lung epithelial cells that surely deserve to be evaluated. More interestingly, GATA3 is not only up-modulated, but it is transcriptional active, as showed by the induction of mRNA transcription of a GATA3 target gene, as *CCL26*. The induction of *CCL26* might modify NK cell status. In particular, it has been shown that CCL26 is able induce the migration of NK cells infiltrated in the epithelial layers of nasal tissue [40]. We hypothesize that SP1 induction of GATA3 and the consequent secretion of CCL26 might induce NK cell migration, as we previously observed (Figure 1A). Further experiments are necessary to prove this hypothesis.

Simultaneously, NK cells showed increased levels of NKG2A/CD94 inhibitory receptor in the presence of SP1 intracellular expression in lung epithelial cells. The percentage of NKG2A-positive cells increased from the 16% to 80%. These NKG2A-positive NK cells were also CD107a negative, supporting the role of this inhibitory receptor in the control of NK cell activation in the presence of SP1. These data are in agreement with the recognized crosstalk between HLA-E and NKG2A/CD94, that induces a higher surface level of HLA-E molecules concurrently with a prevalent expression of NKG2A receptor on the surface of NK cells [15]. Moreover, the maintenance of NK cell activation towards K562 cells, even in the presence of SP1, might be associated to the inability of K562 to express HLA-E molecules [41] and consequently to interact with NKG2A.

The internalization of viral SP1 might induce a cellular stress condition in lung epithelial cells [42], which can result in endoplasmic reticulum stress and consequent down-modulation of classical HLA class I molecules and upregulation of the GATA3 transcription factor. The processing of SP1 by proteasome might create HLA-E-specific peptides that enhance HLA-E surface expression and consequently stimulate NKG2A/CD94 receptors on the surface of NK cells. These aspects deserve further evaluation and more accurate analysis. Further experiments might also elucidate the possible interaction of NK cells with the regulation of adaptative immunity, in particular T cell activation.

These new aspects of interaction between SARS-CoV-2 S1 protein and the host cells might have important implications in the pathogenesis of COVID19, providing opportunities for developing new therapies against SARS-CoV-2. In particular, counteracting the cellular stress, targeting the S1 protein or using the anti-NKG2A monoclonal antibody monalizumab, currently in use for management of rheumatoid arthritis and several neoplastic disorders [43], might represent new anti-SARS-CoV-2 strategies to enhance the innate immune response at the early stage of the disease, inducing mucosal immunity that might lead to a long-term protection against SARS-CoV-2 infection [44].

## Supplementary Materials

The following are available online at https://www.mdpi.com/2073-4409/9/9/1975/s1, Figure S1: (A) Representative dot plots of pre and post NK cell purification from peripheral blood, stained with CD56-PECy7 and CD3-FITC. (B) NK cell viability in the presence of SP1, SP2 from SARS-CoV-2 or spike protein (S) from SARS-CoV. (C) Gating strategy for the analysis of NK cell after the co-culture with K562 cells. Cells were gated for FSC and SSC parameters, selecting singlets and alive cells (7-AAD-). Cell were stained with CD56-PECy7 and CD3-FITC and NK cells were recognized as CD3-CD56+ cells, while K562 cells were CD3-CD56-cells.
