# Supplementary figures and images for: SARS-CoV-2 Spike 1 Protein Controls Natural Killer Cell Activation via the HLA-E/NKG2A Pathway
Source: Cells. 2020 Aug 26;9(9):1975. doi: 10.3390/cells9091975 (PMC7563485; doi:10.3390/cells9091975)

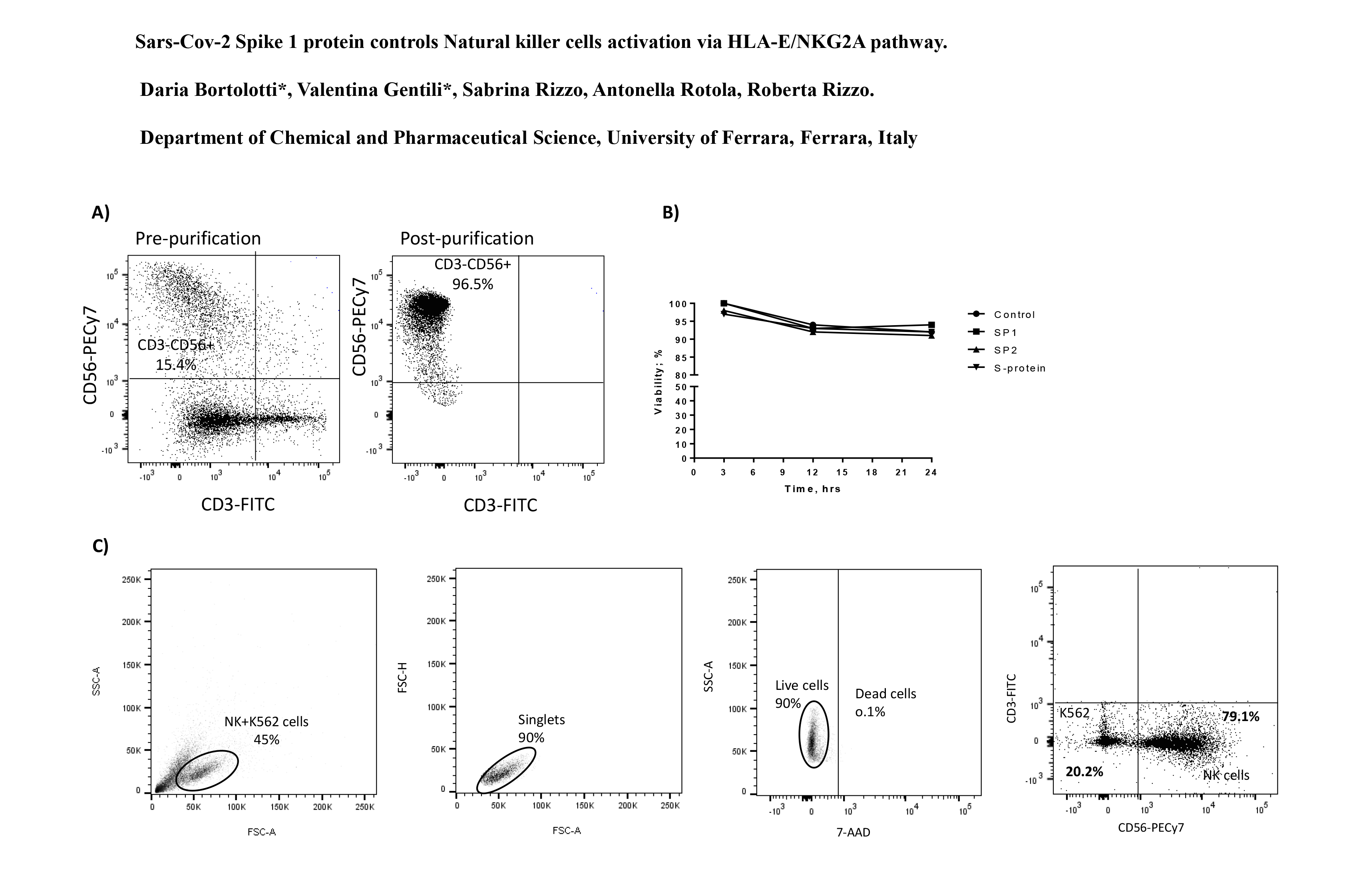

Supplement: Supplementary file 1 [file cells-09-01975-s001.zip › Supplementary Figure 1.tif]
